# Leading Paediatric Infectious Diseases—Current Trends, Gaps, and Future Prospects in Oral Pharmacotherapeutic Interventions

**DOI:** 10.3390/pharmaceutics16060712

**Published:** 2024-05-26

**Authors:** Penelope N. Rampedi, Modupe O. Ogunrombi, Oluwatoyin A. Adeleke

**Affiliations:** 1 Department of Clinical Pharmacology and Therapeutics, School of Medicine, Sefako Makgatho Health Science University, Pretoria 0208, South Africa; ofentse1.sib@gmail.com (P.N.R.); modupe.ogunrombi@smu.ac.za (M.O.O.); 2 Preclinical Laboratory for Drug Delivery Innovations, College of Pharmacy, Faculty of Health, Dalhousie University, Halifax, NS B3H 4R2, Canada; 3 School of Biomedical Engineering, Faculty of Medicine, Dalhousie University, Halifax, NS B3H 3J5, Canada; 4 School of Pharmacy, Sefako Makgatho Health Science University, Pretoria 0208, South Africa

**Keywords:** oral drug delivery systems, infectious diseases, paediatric population, anti-infective drugs, 3D printing, pharmacotherapy, nanoparticulate drug-carriers

## Abstract

Paediatric infectious diseases contribute significantly to global health challenges. Conventional therapeutic interventions are not always suitable for children, as they are regularly accompanied with long-standing disadvantages that negatively impact efficacy, thus necessitating the need for effective and child-friendly pharmacotherapeutic interventions. Recent advancements in drug delivery technologies, particularly oral formulations, have shown tremendous progress in enhancing the effectiveness of paediatric medicines. Generally, these delivery methods target, and address challenges associated with palatability, dosing accuracy, stability, bioavailability, patient compliance, and caregiver convenience, which are important factors that can influence successful treatment outcomes in children. Some of the emerging trends include moving away from creating liquid delivery systems to developing oral solid formulations, with the most explored being orodispersible tablets, multiparticulate dosage forms using film-coating technologies, and chewable drug products. Other ongoing innovations include gastro-retentive, 3D-printed, nipple-shield, milk-based, and nanoparticulate (e.g., lipid-, polymeric-based templates) drug delivery systems, possessing the potential to improve therapeutic effectiveness, age appropriateness, pharmacokinetics, and safety profiles as they relate to the paediatric population. This manuscript therefore highlights the evolving landscape of oral pharmacotherapeutic interventions for leading paediatric infectious diseases, crediting the role of innovative drug delivery technologies. By focusing on the current trends, pointing out gaps, and identifying future possibilities, this review aims to contribute towards ongoing efforts directed at improving paediatric health outcomes associated with the management of these infectious ailments through accessible and efficacious drug treatments.

## 1. Introduction

Infectious diseases have always been one of the main threats to human wellbeing over the long term, and they significantly affect the paediatric population. These diseases often spread quickly and have a high mortality rate especially among children [1,2,3]. Children are more affected than adults by the spread of infectious diseases because their immune systems are still developing, making them more vulnerable [4]. The age range of the paediatric population is 0–18 years old. The International Conference on Harmonization (ICH) divides the paediatric population into several subpopulations, including new-borns (0–27 days), infants and toddlers (28 days–23 months), schoolchildren (2–11 years), and adolescence (12–18 years), based on biological and metabolic changes occurring during development [4,5,6]. An infectious disease is defined as a condition brought on by a particular infectious agent or its toxic by-products and caused by their transmission to a vulnerable host from an infected person, animal, or inanimate source [7,8]. Based on the identity of a pathogen, infectious diseases can be categorised into four major classes: bacterial, fungal, viral, and protozoan infections [9]. They are mainly caused by bacterial or viral illnesses. Among others, severe acute respiratory syndrome (SARS), influenza, pneumonia, and human immunodeficiency virus/acquired immunodeficiency syndrome (HIV/AIDS) are of particular concern [10,11].

When bacteria or viruses cause infection, orally delivered antibiotics or antivirals are usually the treatment of choice. The oral route remains the preferred option for achieving therapeutic effect particularly when it is related to the management of paediatric ailments [12,13,14]. Pharmaceutical formulations designated as oral dosage forms are intended to be consumed via the mouth for absorption and distribution through the digestive system. Orodispersible tablets (ODTs), oral liquid preparations (OLPs), chewable tablets (CTs), dispersible tablets (DTs), oral strips, oral granules, sprinkle formulations, and pellets are some examples of these pharmaceutical formulations. Most oral formulations are in the solid form which makes swallowing and dose regulation more challenging for young children. This factor is significant for treating neonates and infants because liquid formulations are generally advised in these cases [5,15].

Here, we highlight and discuss the current role of oral delivery systems in managing prevalent infectious diseases within the paediatric population and comment on possible gaps to be filled. Concerning the paediatric population, one of the major issues for continued morbidity is the lack of patient compliance. One of the highlights is that children dislike medications administered as injections (intravenous, intramuscular, subcutaneous, etc.) primarily because they are uncomfortable and painful. Furthermore, drug delivery via the injection route requires specialised staff, creating a significant inconvenience to administer treatment at home [16]. They must constantly be hospitalised or travel to specialised facilities to administer such treatment, leading to increased patient non-compliance. Additionally, various drug delivery methods such as nasal, sublingual, buccal, rectal, vaginal, and ocular routes have been reported to pose some administration challenges and may be unsuitable for children, especially neonates and those under 2 years old [17,18]. Oral drug delivery systems (ODDSs), on the other hand, are considered more effective treatment strategies because they are easy to administer, simple, flexible, patient-friendly, painless, non-invasive, and stable [13,14,19,20]. Thus, the oral route remains the most suitable and popular channel of drug delivery for both infectious and non-infectious diseases in children, and it is the focus of the review.

The impact that infectious diseases have on children has been tremendous. This is not only felt by the paediatric population but has also been a considerable burden for the public health sector—ranging from the pressure on pharmaceutical companies to develop suitable and flexible oral dosage forms, to the increase in demand and developing regulations for children to be included in clinical trials, to the hospitals accommodating large numbers of paediatric patients due to the effect caused by these diseases, and to the role of caregivers, guardians, and parents in overseeing their children’s wellbeing [21,22]. This has a domino effect to the extent that it is felt by organisational institutions such as schools, places of worship, parks, and other places where much of the transmission of these infections occur [23]. Furthermore, this has impacted the demand of government providing safe, running water for the children to effectively consume medications outside of hospital set-ups and has increased the demand for health education and access to healthcare services especially in areas occupied by previously disadvantaged individuals and in low-income countries [24]. The burden of these infectious diseases is compounded and requires a multidisciplinary approach to reduce the rates of mortality and morbidity amongst this population set. As such, the importance of this review is to provide the progress made over the past years and highlight the current and future trends as it relates to the management of these infectious diseases using orally delivered medicines.

### 1.1. Search Strategy

Overall, the present manuscript overviews and discusses the role of oral pharmaceutical formulations in the pharmacotherapy of leading paediatric infectious diseases, namely tuberculosis, HIV/AIDS, pneumonia, diarrhoeal diseases (i.e., giardiasis, *Clostridioides difficile*, and *Helicobacter pylori* infections), and respiratory diseases (i.e., influenza, whooping cough, and Group A streptococcus pharyngitis) as mentioned earlier. Since non-parenteral administration routes (e.g., the oral route) present as the most desirable for use by the paediatric population [25], parenteral formulations (e.g., intravenous injections), which may be randomly mentioned, are excluded and outside the scope of this review. Our search was centred on scientific studies and clinical trials published in the English language within the past ten years utilising search terms such as ‘paediatric infectious diseases’, ‘causative pathogens’, ‘oral paediatric formulations for infectious diseases’, ‘oral drug delivery systems’, ‘leading infectious diseases in children’, ‘epidemiology’, ‘transmission’, ‘symptoms’, and ‘treatment of the identified paediatric infectious diseases’. We conducted these searches using accredited academic research databases such as PubMed, Google Scholar, Research Gate, Science Direct, Web of Science, and Scopus.

### 1.2. Leading Infectious Diseases Covered and the Selection Rationale

The diseases discussed herein were chosen based on the classifications made available through governmental agencies and international organisation electronic databases. Specifically, we employed the United Nations International Children’s Emergency Fund (UNICEF), World Health Organisation (WHO), and World Economic Forum (WEF) databases. These organisations listed and classified the most prevalent infectious diseases affecting the paediatric population. The information obtained from the UNICEF database revealed that pneumonia, diarrhoeal diseases, tuberculosis (TB), malaria, and the human immunodeficiency virus/acquired immunodeficiency syndrome (HIV/AIDS) are the most prevalent diseases affecting children, particularly those under 5 years [26]. The WEF also highlighted these same paediatric infectious diseases with the inclusion of influenza [27]. The information contained in the WHO’s database concurred with published data from WEF and UNICEF but with the addition of respiratory diseases (e.g., influenza) [11]. The selected infectious diseases were based on those that caused the highest morbidity and mortality within the paediatric population, and this was founded on the available statistics and those communicable ailments treated with orally delivered drug regimens. All other mentioned diseases that require non-oral drug delivery systems were excluded, such as meningitis, even though they are part of the top ten infectious diseases affecting the paediatric population. Consequently, our screening effort yielded tuberculosis, HIV/AIDS, pneumonia (pneumococcal diseases), and diarrhoeal and respiratory diseases, which then constituted the themes focused on in this submission. As part of our survey, the most prevalent paediatric diarrhoeal diseases, namely giardiasis, *Clostridioides difficile,* and *Helicobacter pylori*, as well as respiratory diseases, specifically influenza, whooping cough, and Group A streptococcus pharyngitis, will be covered. These form the nine leading infectious diseases, managed with predominantly orally delivered drug regimens, contributing to the highest rates of morbidity and mortality amongst children that are discussed here.

## 2. Key Considerations for Paediatric Drug Delivery

The main goal of a drug delivery system is to release the bioactive agent at the correct moment in the right concentration at a specific target site. The physicochemical properties of the therapeutic agent and bio-barriers like the skin and membrane of body organs typically influence the conditions for the successful delivery of drug molecules [25,28,29]. Drug characteristics can differ significantly even when used to treat the same symptoms depending on their molecular size, chemical makeup, hydrophilicity, and capacity to bind a particular receptor. Due to their insolubility in physiological fluids and limited permeability of certain human organs, many drug molecules have insufficient bioavailability [30]. Evidence suggests that the bioavailability of the active pharmaceutical ingredient at the target location influences therapeutic performance in addition to its pharmacological activity [31]. Most drugs used to treat human diseases (e.g., infectious, acute, chronic) during the past few decades have been quick-acting, straightforward substances that are generally in the form of conventional tablets, capsules, creams, liquids, suppositories, injectables, or ointments [28,32]. Additionally, conventional drug delivery systems suffer from severe limitations, such as uncontrolled release, dosage inaccuracy, and repeated application, all of which can hinder patient compliance and the achievement of optimal therapeutic outcomes. To address the drawbacks of current drug delivery techniques, pharmaceutical companies have focused on the development of advanced drug delivery systems. These high-performance, adaptable, and modulated release systems are in high demand due to the major gains in patient compliance, clinical efficacy, increased drug half-life, and economic factors including decreased dosing frequency and administration costs. In contrast to conventional methods, novel delivery systems are meticulously fabricated to improve the performance and distribution of existing drugs. They integrate cutting-edge methods and novel dosage forms to target, regulate, and modulate the distribution of active drug molecules. The efficacy, safety, and patient compliance of a medicine can be significantly increased by switching from a conventional to an innovative drug delivery system to facilitate target site delivery at a pace and amount dictated by the needs of the body [28,33,34,35].

Ideally, every medicine should be created in such a way that the respective patients’ needs are met, and it produces the desired treatment outcome(s) or meets the set therapeutic goal(s) [36]. Designing age-appropriate drug delivery systems for children can be quite daunting particularly because of the significant differences in the developmental phases that they experience which could include continuing growth, quick body transformations, consistently maturing organs, and naturally developing cognitive functions as they advance in age. The presence of these many variables, which must be factored into the formulation development processes, makes designing medicines for the paediatric population relatively complex and challenging [6,37]. The choice of the chemical form of the active drug (e.g., salt, acid, base), its physical appearance (e.g., solid, liquid), type of dosage form, relevant excipients (e.g., taste masking agents, flavours), the need for specialised administration devices (e.g., measuring spoons), and specific administration routes should be carefully decided on during the early stages of pharmaceutical development to ensure that the needs of children within different age categories are appropriately targeted. In addition, the active drug and excipient safety profiles and regulatory approval statuses are important in preventing the exposure of this delicate population to toxic ingredients. Therefore, excipients classified and approved as food grade and/or generally recognised-as-safe are choice options for designing medicines for children. Since solvents can also be key components of paediatric drug delivery systems, clean water is often preferred while the use of organic solvents, especially chlorinate solvents, should be strictly controlled [36,37]. Furthermore, the disease condition to be managed; prescribed duration of treatment; stability considerations and environmental storage conditions; patient acceptability; primary, secondary, and child-resistant packaging; risk of dosing errors; and ease of use by patient and caregiver should all be meticulously investigated and well balanced to meet the unique needs of each child [6,36].

## 3. The Oral Cavity as a Channel for Drug Administration

The administration of drugs occurs in various ways, through different routes in the body, and for different applications. This requires in-depth understanding of the type of drugs administered including how it moves through the body, dosing frequency, possible side-effects/adverse events, the required dosage, and the target site where the drug molecules need to induce the therapeutic effect. Due to the nature of paediatric patients, oral drug delivery is posed as the safest and most convenient option, especially for the treatment of chronic and long-term diseases, but it is not recommended for emergency cases [14,38]. Due to the distinct benefits of the oral route, such as controlled and sustained delivery, ease of administration, feasibility for solid formulations, convenience, patient compliance, flexible dosing, and an enhanced immune response in the case of vaccines, the oral pathway has received the most attention among the different drug delivery methods [39,40,41]. The oral channel of drug delivery possesses a sizable surface area (>300 m^2^ lined with a viscous mucosal layer) that facilitates the adhesion of drugs and subsequent absorption [42]. Typically, drug molecules trapped in mucus are safeguarded from shear forces brought on by flowing gastrointestinal juices [43]. Due to the quantity of enterocytes in various areas of the intestine, particularly the microfold cells (M cells) covering the Peyer’s patches—the lymphoid portion of the small intestine—the epithelium of the human gut is exceptionally absorptive [44,45].

For absorption to occur in the stomach, small intestine, or colon, drugs must be soluble in gastric fluid. The barriers to drug absorption and effectiveness extend beyond those encountered in the gut and include those involving the liver once the drug enters the capillaries beneath the intestinal epithelium. Thus, oral medications cannot be used in emergencies, because of their slow absorption and numerous degrees of barriers they must overcome [25]. Drugs can have local effects in the gut, but most of them are circulated throughout the body via the bloodstream. Drug absorption takes place predominantly in the small intestine because it has a large absorption surface area, which provides added opportunities for drug absorption. In addition, the jejunum and ileum segments of the small intestine’s three major regions (i.e., duodenum, jejunum, and ileum) have a larger capacity for absorption than the duodenum [46]. The average segment length, pH, mucus thickness, drug residence time, and bacterial diversity/population within the various segments are some of the environmental parameters that could influence drug integrity and absorption [47]. The obstacles against oral administration may be broadly classified into biological barriers and technical challenges [46].

Table 1 highlights the different categories of orally delivered pharmaceutical dosage forms, their advantages, and associated limitations.

## 4. Selected Paediatric Infectious Diseases

Diseases caused by infectious microorganisms are common among children in classrooms and other childcare settings. Socioeconomic factors in these settings can increase the likelihood of breakouts in children and adolescents. Certain illnesses can spread directly from one person to another or can be spread by encountering any contaminated environmental sources [63]. Every illness has a unique causative organism and natural course from commencement to conclusion. Many infectious diseases have the potential to spread to other people even when they remain in a pre-symptomatic or subclinical stage without developing into clinical symptoms and indications. Even though they are severe or short-lived, acute infectious illnesses can have serious long-term consequences for public health, such as post-streptococcal glomerulonephritis or rheumatic heart disease. Infections can cause both short- and long-term morbidity. Other infectious diseases, like HIV/AIDS or tuberculosis, are persistent and have their own long-term repercussions [64]. The most prevalent infectious diseases affecting children, as per our earlier-mentioned selection criteria, are discussed subsequently.

### 4.1. Group A Streptococcus Pharyngitis

Group A streptococcus pharyngitis is a significant source of childhood illness in the community. Human infections are brought on by Group A streptococcus (GAS), also sometimes called *Streptococcus pyogenes*, a Gram-positive bacterial pathogen [65]. According to Harris et al., GAS infection is the most typical bacterial cause of acute pharyngitis and accounts for 20–30% of sore throat cases in children. Using conservative estimation methodologies, the WHO reported that globally more than 18 million individuals, including minors, are affected with GAS infections, with an annual increase of more than 1.7 million new notified cases and 500,000 deaths [66]. This makes Group A streptococcus pharyngitis disease the ninth leading cause of death in the paediatric population [67,68,69]. The clinical manifestations of GAS pharyngitis typically include tonsil, impetigo, and pharyngeal inflammation, frequently with patchy exudates and cervical lymph node adenopathy. Other clinical symptoms of GAS pharyngitis include a sudden onset of fever and sore throat. Malaise, headache, nausea, abdominal discomfort, and vomiting are additional typical symptoms [70,71,72]. Person-to-person contact is the most common way for pharyngitis to spread, probably through nasal secretions or saliva droplets from carriers or infected people. As a result, crowded areas have the highest incidence of pharyngitis [67].

Patients with acute Group A streptococcus pharyngitis should be treated with an antibiotic that is likely to eradicate the organism to prevent rare complications (e.g., acute rheumatic fever, and rheumatic heart disease), reduce the length of disease, stop the spread of infection to close contacts, and meet patient needs [73,74,75]. The most effective antibiotic for treating bacterial pharyngotonsillitis in children is penicillin since it is affordable, has a restricted spectrum, and has an established track record of efficacy [76,77]. However, amoxicillin is typically administered as a first-line antibiotic in regions where oral penicillin V is neither produced nor marketed (e.g., Korea) [78]. Generally, patients with penicillin allergy are given first-generation cephalosporins (e.g., cephalexin). Furthermore, the lincosamide antibiotics such as clindamycin, or those belonging to the macrolide class like azithromycin or clarithromycin, are also other effective therapeutic options [78,79,80]. In addition to exhibiting a wider microbiologic spectrum than penicillin V, amoxicillin has a higher oral bioavailability, even when taken with food [79,81]. Furthermore, persons with type 1 hypersensitivity should not be treated with any beta-lactam antibiotics (e.g., cephalosporin, penicillin) [79]. Tetracyclines and trimethoprim–sulfamethoxazole are ineffective options and should not be used to treat GAS pharyngitis in children. Although significant outbreaks of macrolide-resistant strains have emerged in communities, penicillin resistance has not occurred in GAS anywhere in the world, and the baseline level of macrolide resistance is approximately 5–10%. As a result, clinicians should be cognisant of region-specific resistance patterns [73,78].

### 4.2. Human Immunodeficiency Virus/Acquired Immunodeficiency Syndrome (HIV/AIDS)

The human immunodeficiency virus (HIV) is a microbe that preys on the body’s immune system and, if untreated, can cause acquired immunodeficiency syndrome (AIDS). HIV infection makes the infected person more prone to opportunistic infections. Recent statistics showed that about 1.5 million (1.2–2.1 million) children between the ages of 0 and 14 years and 37.5 million (31.8–43.6 million) persons ages of 15 years and above were living with HIV [82,83]. The human immunodeficiency virus infection mostly spreads through bodily fluids like blood. It can spread to children during pregnancy, labour, delivery (childbirth), or breastfeeding [37]. Breastfeeding is responsible for around one-third of all transmissions to young children in communities where it is the norm. Therefore, mother-to-child transmission (MTCT) infection rates among infants are higher in such countries relative to those where HIV-positive women can safely avoid breastfeeding [64]. Contrary to other regions, vertical transmission is the main method of HIV infection in sub-Saharan Africa, where there is a concurrent epidemic in children. African women are disproportionately affected as a result, accounting for 58% of all HIV-positive individuals, with the greatest rate of HIV-positive offspring and the highest rate of AIDS-related deaths [84].

Important limitations include the fact that there are fewer antiretrovirals (ARVs) licensed for use in children than in adults and that there are less liquid dosage forms of these drugs available commercially. Some adult products are capsules, which can be opened, and contents sprinkled on food, and there are also granular dosage forms available for some antiretrovirals that may be adaptable to paediatric dosing. ARVs are also authorised for paediatric usage by extrapolating results from adult data [85,86,87]. In this regard, the coordinated development of safe, effective, and age-appropriate medications and delivery systems continues to be an unmet need in HIV/AIDS research [86,87]. Studies on antiretroviral therapy in children show that several distinct effective ARV regimens result in improvements in morbidity, mortality, and surrogate indicators that are comparable to those attained in adults. When treating HIV infection, highly active anti-retroviral therapy (HAART), which helps to slow the virus’s progression, is recommended, but HAART is unable to destroy the latent virus in isolated and cellular reservoir sites [88]. The two main goals of antiretroviral therapy are virologic control (i.e., decrease in viral load—HIV RNA antigen level) and immune restoration (i.e., restoring CD4 count) [89]. Today’s standard initial treatment, for paediatric patients consists of at least three orally delivered antiretroviral agents targeting different viral proteins. Approved HIV drug classes include (i) nucleoside reverse transcriptase inhibitors (NRTIs), (ii) non-nucleoside reverse transcriptase inhibitors (NNRTIs), (iii) protease inhibitors (PIs), (iv) fusion inhibitors (FIs), and (v) co-receptor inhibitors (CRIs) [90,91]. Two nucleoside reverse transcriptase inhibitors (NRTIs) plus one non-nucleoside reverse transcriptase inhibitor is the recommended first-line regimen for infants and children (NNRTI) [2,92,93].

In expansion to this, the first-line intervention in children is recommended such that when exposed to NNRTIs, treatment based on lopinavir/ritonavir (LPV/r) is administered to patients under the age of three. NVP-based therapy is employed when LPV/r is not an effective choice. The recommended NNRTI for infected children older than three is efavirenz (EFV), with nevirapine (NVP) being the second choice. Abacavir (ABC) + lamivudine (3TC) or zidovudine (AZT) + lamivudine (3TC) should be added to the NRTI regimen for infected children less than three years old who develop tuberculosis (TB) while receiving LPV/r-based treatment, and therapy should be continued until the TB infection is completely cleared out [91,92]. Children between the ages of 10 and 19 years and who weigh 35 kg or more receive the NRTIs with dosing regimens like those of adults [94]. A raltegravir-based regimen may be additionally recommended as the preferred first-line regimen for neonates. Children aged three and older may also receive second-line treatment consisting of one NNRTI and two NRTIs if first-line ARTs are ineffective. The suggested alternative if ABC, Tenofovir (TDF) + 3TC, or Emtricitabine (FTC) fail is AZT + 3TC. The preferred NRTI alternative following the failure of AZT or Stavudine (d4T) + 3TC (or FTC) in first-line therapy is ABC or TDF + 3TC (or FTC) [64,82,95]. The WHO recommends the inclusion of new third-line drugs (e.g., integrase inhibitors and second-generation NNRTIs and PIs) with the lowest chance of developing cross-resistance towards previously used first- and/or second-line ARTs [96].

### 4.3. Pneumococcal Diseases

Pneumonia may be of bacterial or viral origin. *Streptococcus pneumoniae*, a Gram-positive bacterium, is the most prevalent microorganism known to cause community-acquired pneumonia. Respiratory syncytial virus and *Haemophilus influenzae* are also two causative respiratory microbes [97]. *S. pneumoniae* asymptomatically colonises the upper respiratory system and a high colonisation rate has been linked to an increased risk of infection. The colonisation rate is higher in children under 5 years of age than in adults, and it is three times higher in people living in low- and middle-income countries compared to those living in high-income countries [98]. The aspiration of droplets causes pneumococcal infections that can result in pneumonia with or without bacteraemia. *S. pneumoniae* is the second most frequent cause of severe pneumonia after respiratory syncytial virus, accounting for 18.0% of all severe pneumonia infections and 32.7% of all pneumonia-related deaths in children under the age of 5 years old [99]. According to the Global Health Observatory, pneumonia is the most common infectious disease in children, accounting for an estimated 1 million fatalities annually. Recent statistics indicate that more than 800,000 deaths in young children (typically under 5 years of age) are recorded annually, accounting for about 14% of all paediatric deaths globally [26,100,101]. Numerous conditions, including sinusitis, otitis media, pneumonia, bacteraemia, osteomyelitis, septic arthritis, and meningitis, can be brought on by *S. pneumoniae* [102].

The first-line treatment for uncomplicated community-acquired pneumonia in children is beta-lactam antibiotics with subclasses including penicillin and derivatives, cephalosporins, carbapenems, etc. The treatment for community-acquired pneumonia (specifically in patients that are not admitted) is usually amoxicillin [103,104]. Treatment for infants consists of ampicillin/amoxicillin/clavulanate and cefotaxime with imipenem as alternatives. For children between the age group of 3 months and 5 years, the drug of choice is amoxicillin/clavulanate with cefuroxime and/or macrolide as an alternative. For school children from age 5 years and above, amoxicillin/clavulanate and cefuroxime are therapeutic options [105]. For outpatient care, on the other hand, beta-lactam antibiotics (such as amoxicillin, cefuroxime, and cefdinir) are preferred. Most school-aged children can benefit from using macrolide antibiotics (e.g., azithromycin, clarithromycin) to treat pneumococcus and other atypical pathogens. Generally, paediatricians consider azithromycin a better therapeutic option for community-acquired pneumonia as well as lower respiratory tract infections (e.g., acute pharyngitis, acute otitis media) where *S. pneumoniae* is the main cause of illness and mortality. This is because of its favourable side-effect profile, ease of administration, and short treatment period (3–5 days) [106,107,108].

### 4.4. Helicobacter pylori Infections

The presence of the Gram-negative bacterium, *Helicobacter pylori* (*H. pylori*), in the stomach of infected people has been related to the emergence of several gastrointestinal conditions, including chronic gastritis, peptic ulcers, gastric-mucosa-associated lymphoid tissue lymphoma, and gastric cancer [109]. Acute infection can manifest as acute gastritis with nausea or stomach pain. If symptoms are present, they are frequently those of non-ulcer dyspepsia, including stomach pains, nausea, bloating, belching, and occasionally vomiting, if this progresses to chronic gastritis. Most children are asymptomatic but common symptoms can include anorexia, weight loss, vomiting, abdominal pain associated with meals or during the night, and paleness [110]. Although the precise mode of transmission of *H. pylori* is unknown, it is contagious. The most common method of transmission is from person to person through the oral or faecal to oral route [111]. About 50% of the global population is infected with *H. pylori* and children around the world are known to be affected. The prevalence of *H. pylori* infection varies by geographical location with low- and middle-income countries exhibiting noticeably elevated numbers compared to the high-income regions. The overall prevalence of *H. pylori* infection in children globally was estimated to be 32.3% [112].

Antisecretory agents such as proton pump inhibitors (PPIs) and antibacterial drugs are used in combination to treat *H. pylori* infections [113]. The cornerstone treatment for children has long been the traditional triple therapy, which consists of PPIs, amoxicillin, clarithromycin, and metronidazole. Although their precise mechanism of action is still unknown, some researchers have employed alternative compounds, such as phytomedicines, probiotics, prebiotics, and lactoferrin, to improve *H. pylori* eradication rates [114,115]. First-line treatment usually involves the use of PPIs with a combination of two antibiotics and the preferred antibiotics for first-line therapy are metronidazole, amoxicillin, and clarithromycin [116]. According to Lee and Park, the 90% effectiveness rate of clarithromycin-based triple therapy has now dropped to 70–80%. An alternative treatment option for resistance to clarithromycin-based therapy is bismuth-based quadruple therapy [117,118]. A second-line therapy is administered if the first treatment fails and, normally, levofloxacin (or moxifloxacin), amoxicillin, a PPI triple regimen, and bismuth-containing quadruple therapy are used [119]. The evaluation of regimens using fluoroquinolones, including levofloxacin, as second-line therapy in children is limited due to their side-effects in those less than 14 years old [118,120].

### 4.5. Clostridioides (Formerly Clostridium) difficile Infection

*Clostridioides difficile* (*C. difficile*) was first discovered in the gut flora of healthy newborns and, despite being a commensal bacterium, the organism could cause illnesses. According to experts, this is most likely because it produces a toxin that is released into various foods [121,122]. Clinical symptoms of *C. difficile* infection (CDI) range from asymptomatic colonisation to mild diarrhoea and colitis to severe fulminant colitis and possibly fatal toxic megacolon [122]. *C. difficile* is the main cause of antibiotic-triggered diarrhoea and, in children, it is more frequently community-associated. Currently, the incidence of CDI in children has significantly increased with approximately 20,000 cases reported yearly, which could have educational (absence from school) and socioeconomic (parental leave/absence from work) effects. Also, the rate of paediatric hospitalisation due to complicated *C. difficile* infection has also risen by 57% over the past three decades or so [123].

*Clostridioides difficile* infection is often treated using antibiotics, namely metronidazole, vancomycin, and fidaxomicin. Studies have shown that these are safe and effective for use in children. Metronidazole has been demonstrated to be inferior to vancomycin and is only advised for the treatment of mild-to-moderate episodes. Due to the disruption of the natural microbiota after treatment, metronidazole and vancomycin are both linked to high rates of recurrence. Vancomycin and fidaxomicin are comparable in terms of clinical response at the conclusion of treatment, while fidaxomicin is superior in terms of sustained clinical response up to 25 days following the end of treatment [124].

### 4.6. Pertussis (Whooping Cough)

Acute bacterial pertussis is a respiratory infection caused by *Bordetella pertussis* (*B. pertussis*) [125]. A susceptible individual contracts *B. pertussis* from infected persons largely through coming into direct contact with their respiratory secretions or by inhaling the aerosolised droplets of their respiratory secretions. The incubation period is normally 6–10 days but can range from 6 to 21 days. Pertussis, a highly contagious and severe infectious disease, is endemic mainly in low- and middle-income countries and occurs most commonly in unprotected infants younger than 6 months of age and neonates [126]. Neonatal and infant pertussis has the most dangerous clinical presentation, and pneumonia, which occurs in 6% of cases, is the most common complication seen in youngsters. Some of the more severe symptoms include sinusitis, otitis media, superinfections from viruses and bacteria, nutritional deficits brought on by frequent vomiting, and neurologic issues primarily brought on by hypoxia during coughing spells and pain [126,127,128]. Even though increased vaccination rates have significantly reduced pertussis incidence over the past ten years, several affluent countries have recently seen a recurrence of the illness among infants who are too young for vaccination, as well as teenagers and adults [126,128].

Erythromycin, azithromycin, clarithromycin, and trimethoprim–sulfamethoxazole (TMP-SMZ) are commonly used to treat pertussis. In recent years, governmental agencies have favoured promoting the use of newer macrolides (e.g., azithromycin) for treatment as they display higher and persistent intracellular penetration (relative to erythromycin), which may make them particularly effective against pathogens like *B. pertussis* [129]. Azithromycin has been demonstrated to be successful in eradicating *B. pertussis* in 97% of cases after 2–3 days of treatment and in 100% of cases after 14–21 days of therapy [130]. A comparison of erythromycin with azithromycin in the paediatric population showed that both drugs were equally effective in eradicating *B. pertussis*. Clarithromycin is also effective in the treatment of pertussis [129,131,132]. The use of TMP-SMZ fixed-dose combinations is advised in cases of intolerance to macrolides or resistance [130,133]. Other antimicrobial drugs like ampicillin, amoxicillin, tetracycline, chloramphenicol, fluoroquinolones (e.g., ciprofloxacin), and cephalosporins are not recommended in treating *B. pertussis,* due to lack of clinical effectiveness [131,132].

### 4.7. Influenza (Type A and B)

With annual epidemics and infrequent pandemics, influenza is a dangerously contagious respiratory disease that causes morbidity all over the world and kills millions of children and adults. The great degree of variation across influenza species is caused by antigenic shifts and mutations in the genome, which allows for the formation of novel influenza strains and medication resistance [2]. Indeed, seasonal influenza epidemics cause between 250,000 and 500,000 deaths globally each year, with an estimated 1 billion illnesses, of which 3–5 million are severe cases, mainly among the most vulnerable groups like children and the elderly [134]. This respiratory illness is brought on by the influenza A and B viruses in humans. The symptoms can range from mild upper respiratory tract illness with fever, sore throat, runny nose, cough, headache, muscle pain, and fatigue to severe and occasionally fatal pneumonia brought on by the influenza virus or a secondary bacterial infection of the lower respiratory tract. In some instances, influenza infection can result in a variety of other consequences that are not respiratory in nature, thus affecting the heart, central nervous system, and other organ systems [135].

The most popular class of antiviral drugs used for treating respiratory flu-like symptoms are the neuraminidase (NA) inhibitors (e.g., oseltamivir). Oseltamivir, an orally administered NA inhibitor, is widely used globally for the prevention and treatment of influenza A and B in persons one year and older [136,137,138]. The United States Food and Drug Administration also approved oseltamivir for the management of acute, uncomplicated influenza in patients under the age of two weeks [139,140]. Baloxavir marboxil, a cap-dependent endonuclease inhibitor (CENI), is another antiviral drug that works against influenza A and B viruses, including strains that are resistant to oseltamivir. Its uses include the prevention of influenza infections and the treatment of acute, simple cases of the illness. It was approved for use in otherwise healthy individuals ≥5 years old or in patients ≥12 years old who are at high risk of acquiring influenza-related problems [141]. The early administration of oseltamivir in healthy outpatients with uncomplicated laboratory-confirmed influenza showed clinical benefit associated with reduced signs and symptoms of infection, lessened viral loads in respiratory tract tissues, and a lowered likelihood of hospitalisation in children or even adults [138,142,143,144,145]. Early antiviral treatment (within 2 days of illness onset) can decrease the duration of fever and other related symptoms (especially in non-asthmatic children) and can lower the risk of otitis media and respiratory tract complications requiring antibiotics, and hospitalisation in the paediatric population [142].

### 4.8. Giardiasis

This is an intestinal condition that is caused by the flagellated protozoan parasite *Giardia lamblia* (synonym *G. intestinalis* and *G. duodenalis*). According to the WHO, there are almost one billion cases of giardiasis, which may account for 2.5 million annual diarrheal disease fatalities globally. The WHO estimates that 3 billion people live in areas where the prevalence of giardiasis is around 30% [146,147]. Giardiasis is thought to be the cause of 1.2 million illness episodes per year, with a burden of disease comparable to nontyphoidal Salmonella infections and a peak incidence in children 1–9 years of age [148,149,150]. Giardiasis parasite transmission takes place when people consume faeces-contaminated food or water or meet each other [151]. Prolonged diarrhoea, stomach pain, malabsorption, bloating, dehydration, and weight loss are among the symptoms. Frequent intermittent symptomatic or asymptomatic infections are caused by parasites, which are intermittently shed in faeces [150]. Children are more likely than adults to experience severe dehydration because of acute giardiasis, which disrupts daily life and can cause serious health problems [150]. Giardiasis may cause persistent long-term diseases like irritable bowel syndrome after an acute infection [152].

Five-nitroimidazoles (5-NIs), benzimidazole (BIs) derivatives, quinacrine, furazolidone, paromomycin, and nitazoxanide are the six drug classes that make up effective, formally recognised therapeutic agents for giardiasis. Metronidazole, tinidazole, ornidazole, and secnidazole, belonging to the nitroimidazole class, are among the core anti-infective agents that are used to treat *Giardia lamblia* (*G. lamblia*) infection [153,154,155]. Metronidazole is the most often prescribed nitroimidazole antibiotic as, following oral administration, it is promptly and thoroughly absorbed and permeates bodily tissues and fluids such as the saliva, breast milk, semen, and vaginal secretions [155,156]. It is commonly given in two- and three-times-daily doses for 5–10 days or as a short course for 1–3 days in clinical trials [157,158]. Children have been included in many of the trials of both long- and short-course therapy, with outcomes of 80–100% efficacy in the 5–10-day regimens [153]. These schedules are generally well accepted, with the most adverse effects being gastrointestinal distress and metallic taste. A single dose of tinidazole has been shown to produce 80–100% clinical efficacy and noticeable adherence. The paediatric dose (3 years and above) is typically 50 mg/kg (up to 2 g) given as a single dose, while dosing in children younger than 3 years old should be determined by the prescribing physician [158,159]. Secnidazole is typically used as a single dose, just like tinidazole and ornidazole, and the most employed dose is 30 mg/kg for children [153].

Quinacrine, another drug used for treating giardiasis, is quickly absorbed from the gastrointestinal tract and found in all bodily tissues. Metronidazole and furazolidone have been shown to be marginally more powerful than quinacrine, though results vary depending on the in vitro sensitivity testing method employed [158]. Furazolidone, for treating giardiasis, continues to be a crucial therapeutic agent on a global scale and has received approval for usage in children in the United States [154]. Its curative rates of 80–96% have been observed for 7–10-day regimens, even though its effectiveness has typically been thought to be slightly lower than those of metronidazole and quinacrine [154,160]. Albendazole and mebendazole, two drugs from the benzimidazole class, have both been used to treat *G. lamblia* infection, and studies on their efficacy in vivo and in vitro have yielded diverse outcomes [161,162]. They are, however, a viable therapeutic option due to their generally benign side-effect profile and their efficacy against various helminths. The benzimidazoles are not well absorbed from the digestive tract; however, this can be changed by taking them with a fatty meal [161,163]. The aminoglycoside compound, paromomycin, was suggested as a treatment for *G. lamblia* in resistant infections in both children and adults. Paromomycin shows efficacy against *G. lamblia* according to in vitro susceptibility testing, but the activity is typically less potent than that of nitroimidazoles, quinacrine, or furazolidone [147,154].

### 4.9. Tuberculosis

Tuberculosis (TB) continues to be a major cause of sickness and death in children and adults globally [164,165]. TB is one of the oldest infectious diseases known to afflict humans and is brought on by *Mycobacterium tuberculosis* (*Mtb*) [165]. Approximately 7.5 million children are infected with *Mtb*. *Mtb* infection can lead to symptomatic, serious, and transmittable disease (active TB disease) and asymptomatic, non-contagious infection (latent TB). Despite being preventable and/or treatable, more than one million children (under 15 years of age) and half a million adolescents aged 15–19 get sick from TB yearly. Children are particularly susceptible to TB transmission, and it is one of the leading causes of death in the paediatric population with about 205,000 minors dying per annum (excluding HIV-negative children) [165,166,167]. In the case of pulmonary TB (PTB), this usually manifest as a combination of one or more of the following symptoms: extended coughs (often lasting longer than 3 weeks with or without sputum production), coughing up blood, chest pain, loss of appetite, unexpected weight loss, night sweats, fever, and fatigue. In the case of extrapulmonary TB (i.e., TB developing outside the lungs), presenting symptoms will often be dictated by the part of the body affected, although some symptoms such as loss of appetite, night sweat, and fever may be more general [165,168,169].

In general, the principles and drugs used for the treatment of TB (drug-sensitive and -resistant) in children are like those used in adults. Isoniazid (INH), rifampicin (RFP), pyrazinamide (PZA), and ethambutol (ETH) are the first-line drugs for TB treatment. The second line of treatment consists of amikacin, levofloxacin, ethionamide, capreomycin, moxifloxacin, cycloserine, kanamycin, para-aminosalicylic acid, and streptomycin, while the third line of treatment consists of amoxicillin/clavulanate, imipenem, clarithromycin, thioacetazone, bedaquiline, and delamanid [170,171]. The dosing of antitubercular drugs in children is often calculated using body weight [172,173]. Doses extrapolated from adults do not yield equivalent drug exposures in children and consequently produce drug-resistant strains [174,175]. The first-line TB drugs (used more for drug-sensitive strains) are all taken orally, initially for two months with INH, RFP, PZA, and ETH, followed by a four-month continuation period during which only INH and RFP are used, for a total of six months of treatment for active TB [176,177]. These are usually taken as single entities or fixed-dose combinations [176,177,178,179]. The WHO also launched a three-drug regimen (INH, RFP, PZA) for two months followed by two months of INH and RFP which can be used to treat HIV-negative children and young adults with non-severe TB who reside in areas with low HIV prevalence or low INH resistance [177,180]. Children and young adults with non-severe TB who reside in areas with high HIV prevalence rates or high rates of INH resistance receive the standard regimen consisting of INH, RFP, PZA, and ETH for 2 months, and then INH and RFP for an additional 2 months [177,180]. Another factor to consider is that adolescents with TB who are 12 years of age or older can benefit from the 4-month regimen of isoniazid, rifapentine, moxifloxacin, and pyrazinamide (HPMZ), which is now only conditionally recommended by the WHO [177]. Therefore, adolescents between the ages of 12 and 16 have three treatment options: (i) the 4-month HPMZ regimen; (ii) the typical 4-month regimen, with INH, RFP, PZA, and ETH for 2 months and then INH and RFP for 2 months; and (iii) the traditional 6-month regimen—2 months of INH, RFP, PZA, and ETH and 4 months of INH and RFP [177,180].

Moreover, a treatment plan is available for treating drug-resistant tuberculosis (DR-TB) in the paediatric and adult populations [181]. The WHO guidelines recommend choosing injection-free regimens for children and identified three drug groups: group A (levofloxacin/moxifloxacin, bedaquiline, linezolid), group B (clofazimine, cycloserine), and group C (delamanid, ethambutol, pyrazinamide, ethionamide, para-aminosalicylic acid, and amikacin). The treatment plans should include at least four drugs, preferably ones from groups A and B as well as delamanid. Levofloxacin, linezolid, clofazimine, and cycloserine are preferable for use in children under 6 years, while cycloserine is replaced with bedaquiline in children over 6 years. During the intense stage of treatment, a fifth drug may be required in both age groups. The best option among group C medications is frequently delamanid, which the WHO recommends for children over 3 years of age [6,35,177,182].

A summary of commercially available orally delivered pharmaceutical formulations (their doses, treatment duration, and paediatric age ranges) used in the management of the leading infectious diseases in children and adolescents discussed above is presented in Table 2.

## 5. Currents Trends and Gaps

Infectious diseases still largely remain one of the greatest contributors to paediatric mortality and morbidity, particularly those 5 years old and under. The highest mortality rate is associated with diseases such as TB and HIV, whilst the most dominating diseases associated with high morbidity are respiratory and gastrointestinal tract infections. In 2019, the World Health Organisation reported that the cost of illness expended in the African region was about USD 2.4 trillion per annum and, imaginably, it costs more globally [98]. A vast number of challenges must be conquered to effectively manage infectious diseases. The lack of effective drug dosage forms is central to the struggles of treating infectious diseases, and this is further compounded by antibiotic and antiviral resistance, patient-related challenges, erratic drug metabolism patterns (e.g., metabolism by cytochrome P450), drug stability in acidic environments of the gastrointestinal tract, microbe (e.g., bacteria, virus) morphology, the gastric ecosystem, and an increase in bacterial load.

The challenges associated with the use of anti-infective agents are usually linked to the notion that the primary cause of treatment failure against many of these infections is the development of resistance which is mostly caused by the irrational use of these drugs and insufficient bioavailability. Researchers have reported on the existence of multiple occurrences of drug resistance, with attempts to also limit exposure to these anti-infective agents by reducing treatment duration like in the case of pneumonia and *H. pylori*. However, as with most of the infectious diseases, the cornerstone therapy for *H. pylori* in children has long been the traditional triple therapy consisting of the proton pump inhibitors, as well as antibiotics, namely amoxicillin, clarithromycin, and metronidazole that are also known to be the most common amongst drugs employed in the treatment of other bacterial infectious diseases. As such, these anti-infective agents were recorded as one of the most susceptible to microbial resistance [197,198,199,200]. In addition, the World Health Organization published a new recommendation for treating tuberculosis using a first-line regimen to shorten therapy from six to four months in efforts to reduce prolonged exposure whilst maintaining clinical efficacy [201,202]. The patient-associated challenges are also a considerable hurdle, especially for children. The development of dosage forms for the paediatric patients is particularly challenging because of the variations within this population, as children at different developmental stages have diverse physiological attributes. Furthermore, they have difficulties with compliance, acceptability of the dosage form, taste preferences, and concerns about safety for this susceptible patient group. Age-appropriate dosage forms for the paediatric population must be carefully designed and chosen, balancing the target product profile’s quality against development feasibility, technical obstacles, and treatment duration. Children might have a hard time sticking to the prescribed regimens due to all these patient-related challenges, which leads to treatment failure, reoccurrence of these infectious diseases, and possibly drug resistance [15,203].

Another challenge associated with the treatment of these paediatric infectious diseases, both bacterial and viral, is that they mostly require orally delivered medicines, which are typically susceptible to first-pass metabolism, also known as xenobiotic metabolism, in which they are metabolised by cytochrome P450 in the liver before undergoing systemic absorption and distribution. This phenomenon may vary in intensity according to differences in each child’s developmental stage. This may result in a decrease in the bioavailability of the active anti-infective agents. This makes it challenging to treat these diseases as there may be a decrease in effective concentration at the target site, leading to subtherapeutic dosing and unsuccessful treatment. In some instances, this is tackled by increasing the drug dose, which may result in a higher pill burden, possible overdose, unwanted side-effects, and reduced compliance [204,205]. Furthermore, the stability of drugs within the acidic environment of the stomach or susceptibility to enzymatic degradation can hinder optimal drug absorption and pharmacological effect [206]. Whilst there are a lot of physiological barriers that influence treatment success, there is the hurdle of the constant change in morphology of the bacteria and viruses that negatively impact their response to anti-infective agents. This has been probably the largest hurdle in finding a cure for HIV/AIDS as this virus is highly adaptable and is able to change its molecular structure to evade eradication [207]. As viruses such as the influenza virus and HIV can change their morphologies, bacteria can also do the same. Bacteria generally contract and change into microscopic spherical organisms with altered molecular synthesis and gene expression. Different bacterial pathogens have different adaptive mechanisms that allow them to survive the gastric acid, move, and migrate to form biofilms which, in turn, create a barrier for antimicrobial penetration. This is particularly evident in gastrointestinal diseases giardiasis, *Helicobacter pylori*, and *Clostridioides difficile* [118,208,209]. Consequently, it can lead to an increase in bacterial load [210]. All these factors highlight the difficulties experienced with the fight against these infectious diseases and serve as important reasons for why they have been classified as one of the largest contributors to paediatric morbidity and mortality.

Most of the drugs used to treat infectious diseases in children are designed in different forms for oral administration and examples of such include tablets (i.e., chewable, dispersible, orodispersible), capsules, films, sprinkles, granules, liquids-like solutions, syrups, drops, emulsions, and suspensions. These dosage forms are not without their advantages and disadvantages and the form selected for a specific therapeutic application will differ because of factors such as patient age, compliance and convenience, active drug stability in formulation, and clinical setting [50,211]. In paediatric patients, for example, oral dosing is most practical in liquid form such as solutions and syrups [211]. Their main benefit is the guarantee of consistent dose administration because the solutes are evenly distributed throughout the solution. They are considered as the most popular dose form since they are simpler to swallow than pills and capsules. The use of these liquid preparations has significant drawbacks, like chemical, physical, or microbial instability (requiring a preservative), taste issues (requiring flavouring and masking agents), a lack of controlled release properties, a dearth of safe excipients, and susceptibility to inaccurate measured out dose [211].

In addition, solid dosage forms, such as conventional pills, tablets, and capsules, could be too large for children who are known to have swallowing challenges, and this may lead to choking hazards particularly because of their underdeveloped muscular and nervous systems [212,213]. To make these solid dosage forms more usable for the young ones, approaches that split or grind pills into powders have been documented in the past. Fragmenting or manipulating the dosage form can make the administration of the right amount quite challenging, as well as impact on the drug’s stability and effectiveness. Attempting to change the physical form of the solid formulation by simply dispersing a crushed tablet or the content of the opened capsule into an aqueous liquid is often not reproducible or successful, because many solid dosage forms have low solubility in water [214,215].

Due to the limited water solubility of many essential regimen components in both solid and liquid dosage forms, the oral route poses substantial administration issues. As a result, high daily dosages are necessary to obtain a therapeutic concentration in vivo [216]. However, this is even more of a struggle for patients who are diagnosed with long-term chronic diseases such as HIV/AIDS and TB as they have longer treatment regimens, which can last a lifetime like in the case of HIV/AIDS. This is significantly different for gastrointestinal tract diseases (such as giardiasis, *Helicobacter pylori*, *Clostridioides difficile*) and respiratory diseases (like influenza, whooping cough, pneumonia, streptococcus pharyngitis) because they are acute ailments that have short-term treatment regimens typically spanning over 5–14 days. Although oral dosage is the preferred method for patients, it does come with a heavy dose of pills and can leave patients feeling weary, which may make it more difficult for them to follow their treatment plan. Careful adherence to the prescribed course of treatment is essential to prevent the development of viral strains that are resistant to medication and subsequent poor treatment in illnesses like HIV and TB [217,218,219].

Current trends show an increase in the development of orodispersible tablets (ODTs) as there are already commercially available products on the market for the treatment of various infectious diseases. In some cases, when the tablet disintegration or dissolution is sufficiently fast, the use of water can also be avoided [220,221]. ODTs provide a lot of flexibility in drug administration because the tablet can be pre-dispersed in a suitable vessel, put in the mouth right away, or swallowed whole. Due to these, they are accepted by populations of children and newborns. ODTs make it easier to administer and swallow medications, but there is still a lack of dosage flexibility, unlike normal tablets. Additionally, their fragility makes it impossible to divide the pills, which may further limit the dosing flexibility [222,223]. Orodispersible films (ODFs) are comparable to ODTs. ODFs do not require water for delivery, because they quickly disintegrate/dissolve in the oral cavity, which aids swallowing. ODFs also have a classy aesthetic that some patients might like. As opposed to tablets, films have a greater dosage flexibility because varied strengths can be achieved by simply cutting films to the appropriate size [57].

Due to their small size, light weight, and thin form, one of the key drawbacks is the small drug dose that may be integrated, which means that only highly potent drugs are suited for ODF systems. Given some of the disadvantages of ODFs, there are no currently available commercial products on the market dedicated to treating paediatric infectious diseases, including the diseases discussed in this review. However, currently, a few scientific outputs that reported on ODFs designed for loading and delivering anti-infectives include those containing antitubercular agents and ODTs loaded with cefixime trihydrate for infection of the respiratory tract [58,224,225,226].

Other dosage forms of interest include multiparticulates, which are made up of various discrete units like granules, sprinkles, pellets, and/or minitablets. They are anticipated to increase patient acceptance because of their modest sizes and ease of swallowing. Due to their multi-unit makeup, they also provide greater dosing flexibility. Additionally, using film-coating technologies, multiparticulate drug formulations are typically appropriate for achieving controlled drug release and flavour masking, which can also increase patient compliance [57]. The development of these products may be constrained by the gritty mouthfeel that small particles may leave behind, even though they may be easier to swallow and so more acceptable. Although a maximum target size of 2.5 mm has recently been advised, there are also some indications regarding the size and quantity of multiparticulates that are acceptable to patients [227,228]. Before administration, multiparticulates can be mixed in liquids such as milk, juice, water, or apple sauce or placed directly into the patient’s mouth [223]. There are a number of these dosage forms that are approved by the Food and Drug Administrative (FDA) and are commercially available, but they only come in single doses and not as FDCs [179].

Additionally, chewable drug products (e.g., chewing gum, chewable tablets, pharmaceutical gummies) offer positive benefits which primarily aid in the process of swallowing or even circumventing it (in the case of chewing gum dosage forms), as well as water or other fluids not necessary for their administration. Notably, compared to other dose forms, it is their aesthetic qualities that often make them more acceptable or patient friendly. That said, chewable formulations do not allow easy dose adjustments, are not good at concealing flavour/taste, and are typically unable to modulate drug release characteristics [229]. Also, subject-to-subject variability may occur since every patient’s capacity to chew, which differs greatly, directly affects drug release and therapeutic efficacy. The need for mastication could limit the use of chewable dosage forms in the paediatric population. The information that is currently available indicates that chewable tablets are stable and well tolerated in children 2 years and older [230].

Ultimately, most of these formulations are typically considered traditional therapies or conventional drug delivery systems (CDDSs). Poor delivery to the target site is one of the inefficiencies of certain currently available medicines [231]. Most CDDSs have an immediate, high drug release after administration, leading to increased dosing frequency. Misuse is one of the drawbacks associated with increased administration frequency that can lead to drug toxicity. It is also a major challenge for pharmaceutical companies in developing new medicines, as drug solubility in the CDDSs tends to be low, hence affecting efficacy. Moreover, low drug stability is one of the major limitations of using conventional pharmaceutical agents, as the CDDSs in some dosage forms are not sufficient in protecting the active pharmaceutical ingredients against biological fluids in the body and microenvironments [232,233]. However, with the current oral dosage forms developed to improve oral drug delivery, one aspect that is gaining attention is the exploration of gastro-retentive drug delivery systems (GRDDSs). The GRDDSs have gained popularity for oral drug delivery purposes lately and this technology may find use in both adults and children. It is a commonly used method that can address numerous issues with traditional oral delivery, such as poor bioavailability and protecting the drugs from harsh acidic environments by keeping the dose form in the stomach for a long time and releasing the drug gradually [234]. Different innovative approaches like magnetic-field-assisted gastro-retention, high-density systems, mucoadhesion systems, floating systems, and expandable systems are being applied to fabricate GRDDSs [235].

The GRDDSs are designed in such a way that the formulation can float in the gastric fluid due to the low density of such systems, where it may remain for a longer period without changing the pace at which the stomach empties [236]. For example, researchers developed floatable gastroretentive beads of amoxicillin trihydrate, which effectively reduced the growth of *H. pylori* efficiently in vitro and in vivo [237]. Further examples of floating systems that have been explored include antibiotics like clarithromycin [238]. The GRDDSs have also been designed as mucoadhesive systems to extend the residence duration of these delivery systems employing different mucosal routes (topical and systemic), and this has resulted in higher bioavailability, the avoidance of breakdown by digestive enzymes, and first-pass hepatic metabolism [239,240,241]. Scientists have explored the use of these mucoadhesive gastroretentive systems for the delivery of anti-infective agents, and examples include the work of Saifullah and colleagues that detailed the creation of an emulsion-based mucoadhesive self-nano-emulsifying drug delivery system (SNEDDS) to enhance cefixime pharmacokinetics. This proved to be significantly therapeutically effective in both in vitro and in vivo evaluations employing rabbits and could potentially treat Gram-positive and Gram-negative bacteria including *Hemophilus influenza* [242]. Others included a mucoadhesive system containing clarithromycin and amoxicillin (referred to as ‘mucolast’ in this study) for treating *H. pylori* infection which displayed in vivo efficacy in mice [243].

Another gastroretentive delivery approach that has been gaining attention for the encapsulation of anti-infectives covered herein is the application of magnetic systems which can control the movement of a gastroretentive formulation with a small internal magnet by applying a strong magnetic field onto the body surface. Numerous studies highlight the system’s benefits, but the magnet position must be chosen with extreme precision for the system to work [244], and gastroretention may be impacted by the extracorporeal magnet’s intensity [118]. For instance, Abdelaziz and colleagues created vancomycin-conjugated magnetic nanoparticles with specific antibacterial activity by employing a straightforward conjugation technique [245]. Another group fabricated magnetic polymeric stimulus-responsive particles for antimicrobial therapy in the stomach using amoxicillin. This showed good anti-bacterial activity and magnetic field responsiveness that facilitated deep antibiotic penetration into the bacterial mucous layer [246]. Furthermore, gastroretentive expandable systems encapsulating antibiotics were investigated. These systems function by expanding their volume or form to provide a longer gastroretention period. They are designed to be tiny enough to be taken orally without passing through the pyloric sphincter, but expand solely in the stomach, and contract again after the drug release is complete for it to be eliminated from the body. The mechanisms of swelling and unfolding enable volume and shape adjustment, which makes them attractive for use in children. This formulation is sometimes referred to as a ‘plug-type’ system because of its ability to inhibit the pyloric sphincter [247,248]. Reported examples include a swellable, asymmetric, triple-layer tablet loaded with tetracycline, metronidazole, and bismuth to treat *H pylori*. The tablet has a floating property that prolongs the drug’s half-life in the stomach [249,250].

We consider the GRDDSs as novel and potentially useful carriers for the delivery of a variety of anti-infective drugs which could find potential application in paediatric medicines. Although no version of this technology is commercialised, and its efficacy in humans has not been documented, they can potentially function as alternative solutions to the issues associated with the current conventional oral drug delivery systems used for the encapsulation of antibiotics, particularly in the paediatric population.

## 6. Future Possibilities

The current conventional oral drug delivery systems have presented with many drawbacks that include lack of physical and chemical stability, drug degradation, gastrointestinal barriers, bioavailability, solubility, and permeation as stated in this manuscript. These problems require innovative approaches that may be provided using nanomaterials and nanotechnology. Presently, the effects of nanotechnology on humans and animals are opening some of these needed, new research directions and transforming the field of health science and paediatric drug delivery, making it a crucial topic to be taken into consideration as a therapeutic tool [37,251]. There are various nanotechnological techniques that can be explored and incorporated to synthesise novel antibiotic-loaded oral drug delivery systems. In addition, scientists and researchers are constantly trying to invent new delivery strategies capable of enhancing these oral dosage forms by improving their administration, compliance, convenience, and active drug integrity, and reducing side-effects, thus providing them with the potential to cater to the broader paediatric population. Some of these strategies include 3D printing; nipple-shield and milk-based delivery systems; and lipid, polymeric, and metallic formulations. Although many of these suggested strategies are still in the research and developmental phases, most of these applications may make significant contributions to knowledge in managing paediatric infectious diseases. Some of these applications are discussed in further detail within the ensuing subsections.

### 6.1. Three-Dimensional-Printing Technology

Many traditional manufacturing processes are currently being replaced by three-dimensional printing (3DP), also known as additive manufacturing (AM) [50]. Recently, oral drug delivery systems and a dose form for individualised therapy have both been created using 3DP technology. Although the clinical use of 3D-printed drug-eluting devices has not been extensively studied, they allow for customisation in terms of shape, size, and architecture [252]. In the pharmaceutical field, 3DP allows novel drug delivery systems to be manufactured. Single or multiple drugs can be incorporated in one solid dosage form and the release pattern is controlled through the manipulation of the polymeric and non-polymeric excipients. Drug release is controlled using different polymers, geometry, compartmentation, or infill patterns [253]. One would argue that it is difficult to cater for everyone’s preferences, and here is where the technology of 3D printing, which allows for considerable flexibility, comes into play [254]. The ability to customise a dose could make it possible to optimise it by considering factors like gender, age, weight, disease status, needed size, release qualities, duration of use/treatment length, and form. Any type of dosage form, from dissolving tablets to capsules to any form, might be designed, and customised using 3D printing for any ailment. Much research has investigated the use of 3D printing to make medicine for infectious diseases like tuberculosis [255,256]. These studies showed that the formulations displayed the potential to improve drug stability and bioavailability as these are some of the major challenges with existing dosage forms; however, more studies could be conducted to explore pharmacotherapeutic strategies for paediatric infectious diseases, and this avenue holds a promising future to revolutionise medicines for children.

### 6.2. Nipple-Shield and Milk-Based Delivery Systems

Recently, more research efforts have been directed towards the investigation of appropriate vehicles, which will improve the palatability of paediatric formulations. Milk has been explored as a potential vehicle for liquid formulations, making use of its solubilising properties and ability to maintain the stability of the emulsified vehicle and potential milk substitutes to deliver poorly water-soluble drugs by investigating key lipid components that dictate drug solubilisation in milk and infant formulas during digestion [257,258]. Researchers have also explored using the ‘nipple-shield’ as a delivery channel, and this is typically designed to accommodate a drug-loaded insert delivering the active pharmaceutical ingredient into the milk while breastfeeding neonates [259]. The introduction of this delivery system has found some use in the flexible administration of antiretroviral drugs for the prevention of the mother-to-child transmission of HIV. Additionally, it will make it easier to deliver medicines to neonates who are often not catered for in the making of medicines. In our opinion, this delivery approach could potentially be employed for the delivery of medicines indicated in the treatment of other leading paediatric infectious diseases, particularly in neonates. Another interesting and growing area are baby bottles coupled to graduated syringes for facilitating the administration of liquid formulations. Others include modified pacifiers and the ‘dose sipping syringes’, which can be used either as a conventional oral syringe or as a straw for the administration of anti-infectious agents formulated as liquid dosage forms [260,261]. These are not commercialised yet, because they can potentially increase the overall product costs, but nonetheless, they could function as alternative strategies for administering medicines to children that is worth exploring.

### 6.3. Lipid-Based Nanoformulations

One of the key demerits of liquid drug carriers as it relates to patient acceptance is the lack of controlled release kinetics, which typically require the administration of multiple doses throughout the day [57,262]. One of the ways to combat this issue is using nanomedicine because drug molecules can be embedded into these unique carrier matrices or even modified to produce delivery systems with such properties, setting them apart from conventional drug products [263,264]. There are various applications to nanomedicine, and one way is to utilise lipid-based nanoformulations for the oral administration of bioactive agents that are not well solubilised in water, such as BCS classes II and IV drugs, which have been employed. These formulations make up about 3% of all the drug products on the market and a few of them are indicated in the treatment of infectious diseases covered here (e.g., Lopinavir/Ritonavir marketed as Kaletra^®^) [13]

Lipid-based systems can be further divided into lipid solutions, lipid suspensions, emulsions, multiple emulsions, micro- and nanoemulsions, self-emulsifying and self-micro-emulsifying systems, solid lipid nanoparticles, solid lipid dispersions, niosomes, and liposomes based on their composition, size, and chemical properties. Lipid-based formulations are present as a viable option for oral delivery due to their inherent biocompatibility, diversity in particle size, ability to scale up, and cost-effectiveness. Most of these formulations can be ingested orally as solid dosage forms containing liquids in it or as solutions or suspensions. Additionally, these formulations can support rapid or sustained drug release [13]. The production of sustained-release liquids has been studied using a variety of methods, including ion exchange resins, coated microparticles in suspension, and drug microemulsions, among others. Azithromycin extended-release (first extended-release suspension) oral suspension is an example of a sustained-release liquid formulations on the market [260].

### 6.4. Polymeric Nanoformulations

Polymeric formulations can also be used in the development of innovative oral delivery systems as they have gained exponential growth in the field of nanotechnology. With their improved drug loading capacity, increased blood circulation time, simple chemical modification, and convenient surface functionalisation, polymer-based nanoparticulate platforms have been extensively researched for various treatments [264]. Drugs may be hydrophobically loaded into polymeric micelles, covalently conjugated into polymers, or encapsulated in polymeric nanoparticles. Oral drug delivery systems have been produced using a variety of natural and synthetic polymers. Dextran, chitosan, gelatine, and alginate are some examples of common natural polymers, and polylactide-coglycolide (PLGA), polylactide (PLA), polycaprolactone (PCL), polyglycolide, polycyanoacrylate, polymethylmethacrylate (PMMA), poly(propyleneimine) (PPI) dendrimers, and polyaziridine are examples of synthetic polymers used in oral drug delivery [265]. Without chemical conjugation, drugs could be loaded or encapsulated into polymeric nanoparticles. Insoluble medications can be delivered using polymeric nanocarriers, which can also be used to target the drugs to specific parts of the GI tract, reduce the impact of food on drug absorption, make it easier for drugs to pass the mucosal barrier, and enable receptor-mediated intracellular drug administration [266].

### 6.5. Metallic Nanoparticles

When creating antibacterial formulations, the use of metal nanoparticles is a typical strategy. The most studied metals in medicine are gold and silver nanoparticles, which offer a wide range of potential uses. Pure metal particles like zinc, gold, and silver are used to create metallic nanoparticles. These particles have unique surface charges and hydrophobicity and they have been shown to have stronger antibacterial properties [267]. Metallic nanoparticles have been reported to be good anti-agents against infection-causing pathogens that have become resistant to antibiotics, have adapted to the gastric environments, and cause biofilms that delay the permeation of antibiotics [268]. For example, according to Gopinath and collaborators, gold NPs made from *Tribulus terrestris* fruit extract had bactericidal effects on *H. pylori* [269]. Ortiz-Benitez et al. also attempted a first-of-its-kind study to investigate and explain how gold nanoparticles destroy the bacterium *Streptococcus pneumoniae* [270]. Choi et al. have also highlighted the use of gallium nanoparticles to inhibit the growth of *Mycobacterium tuberculosis* and HIV [271]. Through the advancement of technology, it can be a future possibility to incorporate metals with antibiotics to create medicines that have a dual effect for use in children and adolescents. There are, however, potential challenges posed by the exposure to metallic nanoparticles. The small sizes of nanoparticles give them the ability to permeate physiological barriers of living organisms, causing harmful biological reactions and can be toxic to the brain and cause lung inflammation and cardiac problems. In fact, certain nanoparticles have been found to cause permanent cell damage through organ injury and oxidative stress, due to their size and composition. The level of toxicity of nanoparticles is suggested to be dependent on factors such as composition of the nanoparticle, size, surface functionality, crystallinity, and aggregation. Moreover, the toxicity of a nanoparticle in an individual is dependent on the genetic make-up of that individual, which is determined by the individual’s ability to adapt and respond to toxic substances. For children, this may pose more risk as their bodies are constantly undergoing physiological changes (continuously developing), which could make it quite challenging for their bodies to tolerate exposure to these metallic particles [272,273,274].

## 7. Concluding Remarks

The prevalence of infectious ailments has proven to be a difficult task to address. It is still one of the largest contributors to a high disease burden in the paediatric population. This is due to several compounded challenges that include antibiotic and antiviral resistance, patient- and caregiver-related problems, the rapid metabolism of drugs by cytochrome P450, the stability of drugs in acidic environments (e.g., stomach), the changing morphology of microbes, the gastric ecosystem, and an increase in bacterial load plus the lack of effective antimicrobial dosage forms as the core of the struggles associated with treating these infectious diseases. There have been several trends over the last decade that have explored challenges associated with oral anti-infective agents, and what we have observed, in recent years, is that the pursuit of formulating oral dosage forms is leaning more towards creating additional solid pharmaceutical formulations. This encompasses the incorporation of orodispersible, and dispersible tablets as more flexible drug formulations needed to cater more for the paediatric population and move away from liquid dosage forms as they are less physically and chemically stable, display erratic bioavailability, and increase administration volumes and errors. In addition, there is an increase in the exploration of gastroretentive delivery systems such as 3D-printed solid formulations; floatable systems; expandable carriers; and mucoadhesive, high-density, and magnetic systems to preserve the integrity of tablets, capsules, and other solid pills. All these approaches attempt to improve issues around gastrointestinal barriers, solubility, permeability, bioavailability, and stability that conventional, orally administered anti-infective agents are facing. Although there are various studies on these described systems, many of them have not been translated into the clinical settings. Even with the new trend of moving to developing more solid dosage forms, there are, however, very few drug products that are commercially available for managing paediatric infectious diseases. In our opinion, all of these could provide researchers with more opportunities for in-depth investigations and the innovation of more practical and efficient oral anti-infective drug delivery strategies for children and adolescents. Other potentially useful explorations include the fabrication of improved orally delivered dosage forms employing novel technologies like 3D-printing, nipple-shield, and milk-based delivery systems. In addition to these avenues are nanomedicines (e.g., polymeric, lipid-based, and metallic variants) that can serve as effective oral antibiotic delivery systems for easier consumption and improved compliance in the paediatric population and their caregivers, thus possibly addressing major drawbacks of traditional formulations. One of the key aspects of successfully treating these infectious diseases depends heavily on completion of the treatment course to induce therapeutic efficacy, limit the development of drug resistance, and reduce the prevalence of these diseases, and as such, explorations of new research avenues need to be given attention to fulfil this objective.

## Figures and Tables

**Table 1:** Orally delivered pharmaceutical dosage forms.

Dosage Form	Distinguishing Characteristics	Limitations	References
**A.** **Oral solid formulations**			
Solid tablets (mini, soft, scored)	For immediate or modified release (gastro-resistant, delayed, extended, protracted release kinetics), and tablets can be coated or uncoated.	Young children are unable to swallow pills whole. Higher doses require many minitablets, but tolerability has been acceptable. Poor dose flexibility.	[48]
Chewable tablets	Immediate-release tablets can be chewed, crumbled, or broken without exerting any discernible effects on the stability and bioavailability of the active drug.	Taste may be drastically altered. Bioavailability may be altered depending on chewing ability.	[49]
Capsules	Capsules help to mask the unpleasant taste of its contents and the drug has limited interaction with the excipients. They are good for hydrophobic drugs and oily active substances that are suspended or dissolved in oil.	Some might also be opened, but this action is more likely to affect bioavailability. Young children are unable to swallow the dosage form whole.	[50]
Sprinkles	Can be used in neonates and seriously ill infants. Can be taken with foods and drinks to improve palatability.	Ability to swallow food or fluid substances (containing drug formulations) is needed. Compatibility with food/drinks.	[51]
Gummy formulations	Ease of administration, safety, and lack of stability challenges for dosage formulations. Soft, elastic, springy, and flexible. The enhancement in flavour, fragrance, and texture can stimulate salivation, making swallowing easier.	Without adding a lot of sweets and flavourings, it could be difficult to include medications with strong or disagreeable tastes—like bitterness—into gummy formulations. They also require airtight storage in a dry environment due to their hygroscopic nature. These formulations have reportedly been linked to cases involving tooth damage or denture rupture.	[14,52,53]
**B.** **Formulations manufactured as solids but consumed as liquids**			
Powders for reconstitution	Due to the absence of the aqueous vehicle, reconstitution formulation lightens the final product’s weight, potentially lowering transportation costs. Avoiding the physical stability issues that conventional suspensions frequently have.	The integrity of a drug is influenced by several physical aspects of the dosage form, including storage temperature, formulation sedimentation rate, and liquid flow characteristics like viscosity, pourability, dispersion, flocculation, and content homogeneity. It is challenging to prevent the deterioration of powders that contain hygroscopic, deliquescent (tend to melt or dissolve in a humid atmosphere), or fragrant materials.	[35,54,55]
Effervescent tablets	Excellent dose flexibility. Guarantees active ingredient. Stable until dissolution and administration.	Handling friable and brittle.	[48]
Orodispersible tablets, strips, and films	Designed to dissolve in the mouth in a matter of seconds. Orodispersible tablets (ODTs) eliminate the need to swallow the tablet whole. They provide a great deal of flexibility in terms of administration because the tablet can be pre-dispersed in an appropriate vehicle, administered straight into the mouth, or even completely swallowed, depending on preference. Films and strips have greater dosage flexibility because varied strengths can be achieved by simply cutting films/strips to the appropriate size.	They make medications easier to administer and swallow but they do not offer the same degree of dosing flexibility as traditional tablets, necessitating the use of a range of dosage strengths to meet the needs of all populations. Orally disintegrating formulations with unpleasant-tasting active pharmaceutical ingredients would require taste and flavour masking because the medicine becomes exposed to the patients’ taste buds within the mouth. Sweeteners and flavours are typically added to the recipe to improve palatability. Time spent in the mouth may affect the drug’s bioavailability. They are usually friable and brittle, so they are quite challenging to handle.	[56,57,58,59]
Granules	Infants and young children can swallow powders and grains easily. Stability, portability, good dosage uniformity. Options for different doses and modified release.	Children may not enjoy the way they feel in their mouths.	[49]
**C.** **Oral liquid formulations**			
Oral drops and solutions	Easy to swallow.	For this dosage type, the effectiveness of the dose-measuring equipment is crucial.	[60]
Suspensions and syrups	In some circumstances, using an API in suspended form can help mask unpleasant taste and flavour and thus make swallowing easier.	Resuspendability should be a stability criterion since, in some cases, caking or sedimentation of the suspension during storage may pose a major risk for dosing errors. Healthcare professionals must make sure that children will receive a dosing device that is suitable to deliver the recommended dose and that any inappropriate devices are removed from the packaging because formulations may be marketed for a broad patient population without a dosing device or with a device that is specific for certain doses. The drug substance may be chemically unstable in the aqueous vehicle.	[61,62]

**Table 2:** Commercially available oral drug formulations employed in the pharmacotherapy of the leading paediatric infectious diseases discussed herein.

Disease	Drug	Oral Formulation	Age Range	Dosage and Duration	References
**Group A streptococcus pharyngitis**	Penicillin V	Oral solutions, tablets, capsules, reconstitutable suspensions	1 month–12 years	250 mg/kg (6–12 h) for 10 days	[79,183,184]
			0–18 years	250 mg/kg (6 h) for 10 days	
	Amoxicillin	Reconstitutable suspensions, liquid suspensions	0–18 years	50 mg/kg four times a day for 10 days	
	Cephalexin	Reconstitutable suspensions	≥3 years	40 mg/kg/day twice a day for 10 days; max dose 500 mg	
**Pneumococcal diseases**	Azithromycin	Tablets, liquid suspension, oral reconstitutable suspension	≥6 months	10 mg/kg/day for 5 days	[105,185,186,187]
	Clarithromycin	Tablets, oral reconstitutable suspensions		15 mg/kg/day for 10 days	
	Amoxicillin	Liquid suspensions	≥3 months	90 mg/kg/day for 5–10 days	
	Clavulanate	Tablets, oral suspensions	≤3 months ≥40 kg	6–13 mg/kg (500 mg twice a day for 10 days)	
***Helicobacter pylori***	Clarithromycin	Liquid suspensions, oral solutions	15–24 kg	750 mg twice a day for 14 days	[116,188,189]
			25–34 kg	1000 mg twice a day for 14 days	
			≥35 kg	1000 mg twice a day for 14 days	
	Amoxicillin	Reconstitutable suspensions	15–24 kg	500 mg twice a day twice a day for 14 days	
			25–34 kg	750 mg twice a day for 14 days	
			≥35 kg	1000 mg twice a day for 14 days	
	Omeprazole	Tablets, capsules, oral suspensions	15–24 kg	20 mg twice a day for 14 days	
			25–34 kg	30 mg twice a day for 14 days	
			≥35 kg	40 mg twice a day for 14 days	
***Clostridioides difficile***	Metronidazole	Tablets, capsules, oral suspensions		25–30 mg/kg/day for 14 days	[190,191]
	Vancomycin	Capsules, oral solutions		30–40 mg/kg/day	
	Fidaxomicin	Tablets, oral suspensions	>6 months	200 mg twice a day for 10 days	
**Whooping cough (Pertussis)**	Erythromycin	Tablets, powders, liquids	1–5 months	40–50 mg/kg four times a day for 14 days	[192]
			≥6 months	40–50 mg/kg twice a day for 14 days (max of 2 g per day)	
	Azithromycin	Suspensions, tablets, capsules	≤1 months	10 mg/kg/day for 5 days	
			1–5 months	10 mg/kg/day for 5 days	
			≥6 months	10 mg/kg day 1 and 5 mg/kg for day 2–5	
**Influenza type A and B**	Oseltamivir	Capsules, reconstitutable suspension	0–8 months	3 mg/kg/dose for 5 days	[141,193]
			9–11 months	3.5 mg/kg/dose for 5days	
			1–12 years	>40 kg 75 mg per day for 5 days	
	Baloxavir	Tablets	40–79 kg	40 mg for 5 days	
			≥80 kg	80 mg for 5 days	
**Giardiasis**	Quinacrine	Tablets, capsules	0–12 years	2 mg/kg three times a day for 5–7days	[194]
			13–18 years	100 mg three times a day for 5–7 days	
	Metronidazole	Tablets, capsules, oral suspensions	1–3 years	100 mg four times a day for 5–7 days	
			3–7 years	800 mg four times a day for 5–7 days	
			7–10 years	1000 mg four times a day for 5–7 days	
			>10 years	400 mg three times a day for 5 days	
	Tinidazole (Off-label)	Tablets	>3 years	50–60 mg/kg four times a day for 3–5 days	
	Secnidazole	Tablets, oral granules	>12 years	30 mg/kg once. Based on response	
	Ornidazole	Tablets	≤35 kg	40 mg/kg single dose for 2 days	
			>35 kg	1500 mg single dose for 2 days	
	Albendazole	Chewable tablets	6–12 years	400 mg four times a day for 5 days	
	Mebendazole	Chewable tablets	5–15 years	200 mg three times a day for 3 days	
	Nitazoxanide	Tablets, oral suspensions	1–3 years	200 mg/day divided twice a day for 3 days	
			4–11 years	400 mg/day divided three times a day for 3 days	
			≥12 years	1000 mg/day divided twice a day for 3 days	
	Paromomycin	Capsules	Not clearly stated	25–30 mg/kg/day three times a day for 10 days	
	Furazolidone	Tablets, oral suspension	>1 m	400 mg three times a day for 7–10 days	
**Tuberculosis**	Isoniazid	Tablets, syrup	Based on weight	10 mg/kg max of 300 mg for 3 times a week for 2 months	[195]
	Rifampicin	Capsules, oral suspension	Based on weight	15 mg/kg max 600 mg/day twice weekly for 2 months	
	Pyrazinamide	Tablets	Based on weight	35 mg/kg max of 900 mg/day for 2 months	
	Ethambutol	Tablets	Based on weight	range 15–25 mg/kg for 2 months	
**HIV/AIDS**	Efavirenz	Tablet	10 to ˂14 kg	200 mg daily	[92,196]
			>14 to ˂25 kg	250–300 mg daily	
			>25 to <40 kg	350–400 mg daily	
			≥40 kg	600 mg daily	
	Abacavir/ Lamivudine	Orodispersible tablet (60 mg/30 mg)	3 to ˂6 kg	2 tablets daily	
			6 to ˂10 kg	3 tablets daily	
			10 to ˂14 kg	4 tablets daily	
			14 to ˂20 kg	5 tablets daily	
			20 to ˂25 kg	6 tablets daily	
		Orodispersible tablet (120/60 mg)	3 to ˂6 kg	1 tablet daily	
			6 to ˂10 kg	1.5 tablets daily	
			10 to ˂14 kg	2 tablets daily	
			14 to ˂20 kg	2.5 tablets daily	
			20 to ˂25 kg	3 tablets daily	
	Atazanavir	Capsules (100 mg)	10–25 kg	2 tablets daily	
		Capsules (200 mg)	10–25 kg	1 tablet daily	
	Dolutegravir	Dispersible tablet 5 mg	3 to ˂6	1 tablet daily	
			6 to ˂10	3 tablet daily	
			10 to ˂14	4 tablet daily	
			14 to ˂20	5 tablet daily	
			20 to ˂25	6 tablet daily	
		Dispersible tablet 10 mg	3 to ˂6	0.5 tablet daily	
			6 to ˂10	1.5 tablet daily	
			10 to ˂14	2 tablet daily	
			14 to ˂20	2.5 tablet daily	
			20 to ˂25	3 tablet daily	
	Zidovudine	Dispersible tablet	>4 weeks	60 mg daily	
		Oral liquid	3–14 kg	10 mg/mL daily	
	Abacavir	Dispersible tablet	>4 weeks	60 mg daily	
		Oral liquid	>3–14 kg	20 mg/mL daily	
	Lamivudine	Oral liquid	3–14 kg	10 mg/mL daily	
	Lopinavir/ritonavir	Tablet	>10 kg	100 mg/25 mg daily	
		Oral Pellets	>3 kg	40 mg/10 mg daily	
		Oral Granules	>3 kg	40 mg/10 mg daily	
		Oral solution	>3 kg	80 mg/20 mg/mL daily	
	Raltegravir	Oral granules for suspension	>4 weeks	10 mg/mL daily	

Note: This table only represents standard orally administered formulations that are recommended for the treatment of infectious diseases discussed in this submission. This treatment can be taken in conjunction with other drugs for various reasons such as managing side-effects that may arise, patients presenting with comorbidities, different disease stages and manifestations/symptoms, nutrition, stage of pregnancy (e.g., pregnant teenagers), allergic reactions, marketed and/or lack of available drugs from different countries, and diverse dosage forms.
